# Consequences of Alcohol Use in Diabetics

**Published:** 1998

**Authors:** Nicholas V. Emanuele, Terrence F. Swade, Mary Ann Emanuele

**Affiliations:** Nicholas V. Emanuele, M.D., is a professor in the Department of Medicine, the Division of Research on Drugs of Abuse, and the Molecular Biology Program and director of the Division of Endocrinology and Metabolism at Loyola University Stritch School of Medicine, Maywood, Illinois, and a staff physician at the Veterans Affairs Hospital, Hines, Illinois. Terrence F. Swade, M.D., is in the private practice of endocrinology in the Chicago, Illinois, area. Mary Ann Emanuele, M.D., is a professor in the Department of Medicine, the Department of Molecular and Cellular Biochemistry, and the Division of Research on Drugs of Abuse, Loyola University Stritch School of Medicine, Maywood, Illinois

**Keywords:** AODE (alcohol and other drug effects), diabetes, glucose, insulin, pancreas, heavy AOD use, adverse drug effect, disease complication, nutrient intake, ethanol metabolism, hypoglycemia, ketoacidosis, lipid metabolism, triglycerides, cholesterol, pathologic process, cardiovascular disorder, neuropathy, psychosexual dysfunction, visual system disorder, literature review

## Abstract

The hormone insulin, which is produced in the pancreas, is an important regulator of blood sugar levels. In people with diabetes, the pancreas does not produce sufficient insulin (type 1 diabetes) or the body does not respond appropriately to the insulin (type 2 diabetes). Alcohol consumption by diabetics can worsen blood sugar control in those patients. For example, long-term alcohol use in well-nourished diabetics can result in excessive blood sugar levels. Conversely, long-term alcohol ingestion in diabetics who are not adequately nourished can lead to dangerously low blood sugar levels. Heavy drinking, particularly in diabetics, also can cause the accumulation of certain acids in the blood that may result in severe health consequences. Finally, alcohol consumption can worsen diabetes-related medical complications, such as disturbances in fat metabolism, nerve damage, and eye disease.

Diabetes mellitus, which affects an estimated 16 million people in the United States, is a complex disorder interfering with the body’s sugar (i.e., carbohydrate), fat (i.e., lipid), and protein metabolism. The disease is caused in most cases by a deficiency or complete lack of the hormone insulin, which is produced in the pancreas, or by an inability of the body to respond appropriately to insulin (i.e., insulin resistance). The results of both conditions can include chronically elevated blood sugar levels, excessive excretion of sugar in the urine, and the accumulation of certain acidic substances in the blood. If not prevented or treated properly, these changes can lead to coma and even death. Other adverse events associated with diabetes affect the eyes, kidneys, nervous system, skin, and circulatory system.

Because alcohol use, at least on a social level, is widespread among diabetics as well as nondiabetics, clinicians and researchers must understand alcohol’s effect on the progression and complications of diabetes. This article first reviews the pathophysiology of the two major forms of diabetes, type 1 and type 2. It then summarizes the current state of knowledge regarding alcohol’s effects on blood sugar regulation and other aspects of metabolism as well as on the cardiovascular, neurological, and eye complications associated with the disease.

## Pathophysiology of Diabetes Mellitus

The two most common forms of diabetes are type 1 and type 2 diabetes, with type 2 diabetes accounting for at least 90 percent of all cases. Type 1 diabetes is an autoimmune disease—that is, a disease in which the body’s immune system attacks and destroys not only foreign molecules or organisms but also some of the body’s own cells. In most patients, the disease develops before age 40, primarily during childhood or adolescence. In those patients, the immune system attacks certain cells of the pancreas, called beta cells. (For more information on the structure and function of the pancreas, see textbox, p. 213.) Beta cells produce insulin, one of the two major hormones involved in regulating the body’s blood sugar levels and other metabolic functions. Most importantly, insulin leads to the uptake of the sugar glucose into muscle and fat tissue and prevents glucose release from the liver, thereby lowering blood sugar levels (e.g., after a meal) (see figure). As a result of the immune system’s attack, the beta cells can no longer produce insulin. Consequently, the patient essentially experiences total insulin lack. Because insulin is a key metabolic hormone, insulin deficiency leads to major impairment of the body’s regulation of carbohydrate, lipid, and protein metabolism.

**The Pancreas and Its Hormones**

The pancreas, which is located behind the stomach, serves two functions. The first function, which involves most of the pancreatic cells, is the production of digestive enzymes. Those enzymes are secreted directly into the gut to ensure effective food digestion. The second function is the production of several hormones. Two of the hormones (i.e., insulin and glucagon) are potent regulators of blood sugar levels. Both hormones are produced in areas of the pancreas called the Islets of Langerhans, which, quite literally, are “islands” of hormone-producing cells in a “sea” of digestive enzyme-producing cells. Among other cell types, the Islets of Langerhans include an inner core of insulin-producing beta cells surrounded by a layer of glucagon-producing alpha cells.

Insulin primarily serves to lower blood sugar levels by promoting the uptake of sugar (i.e., glucose) in the muscles and fat (i.e., adipose) tissue as well as the conversion of glucose into its storage form, glycogen. In addition, insulin inhibits the production of more sugar molecules (i.e., gluconeogenesis) in the liver. Conversely, glucagon primarily serves to increase blood sugar levels. Accordingly, it promotes gluconeogenesis and the breakdown of glycogen into glucose. The actions of insulin and glucagon must be finely balanced, because both lower than normal blood sugar levels (i.e., hypoglycemia) and higher than normal blood sugar levels (i.e., hyperglycemia) can have deleterious effects on the body.

Type 2 diabetes, which in most cases develops in people over age 40, has a somewhat different pathophysiology than type 1. People with type 2 continue to produce insulin in early disease stages; however, their bodies do not respond adequately to the hormone (i.e., the patients are resistant to insulin’s effects). Thus, insulin does not lower blood sugar levels to the extent that it does in people without diabetes. The insulin resistance is partly inherited and partly acquired. For example, obesity, inactivity, and cigarette smoking may worsen genetically determined insulin resistance.

Insulin resistance does not immediately lead to overt diabetes, because the patient’s pancreatic beta cells initially can increase their insulin production enough to compensate for the insulin resistance. In fact, insulin-resistant people have higher than normal insulin levels (i.e., are hyperinsulinemic^1^). In time (i.e., probably after several years), however, the pancreas cannot keep up with the increased demand for insulin; although insulin production still may be higher than in nondiabetic people, it is no longer sufficient to overcome insulin resistance. Ultimately, insulin secretion declines even further, to levels below those seen in nondiabetics (although generally still higher than those seen in type 1 diabetics). At that point, when a deficit in insulin secretion is combined with a state of insulin resistance, the person develops type 2 diabetes. Thus, whereas type 1 diabetes is characterized by a complete lack of insulin production, type 2 is characterized by reduced insulin production plus insulin resistance. The reasons underlying defective insulin secretion and insulin resistance, which are still under investigation, are complex and beyond the scope of this article (for a review, see DeFronzo 1997).

**Figure:**
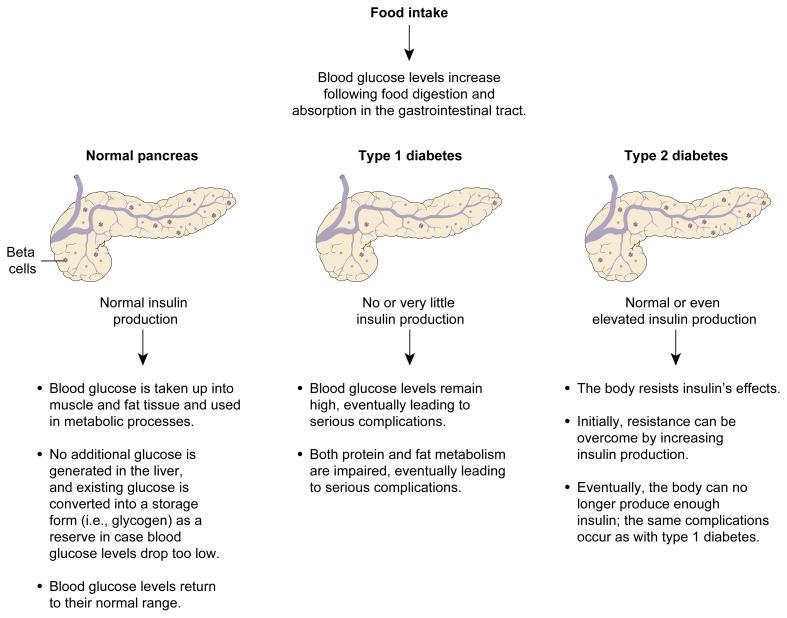
Blood glucose regulation by insulin in healthy people and in people with type 1 or type 2 diabetes.

People with either type 1 or type 2 diabetes generally are treated with insulin injections or—for people with type 2—with medications that stimulate the body’s own insulin production (e.g., a class of medications called sulfonylureas or an agent called repaglinide). Those medications, however, may lead to lower than normal blood sugar levels (i.e., hypoglycemia): As the medications enhance insulin production, the body responds to the increased insulin levels by increasing glucose uptake into the muscle and fat tissue and decreasing glucose secretion from the liver. As a result, the body’s hormone system mounts a counter-regulatory response: It secretes an array of hormones that through various mechanisms raise blood sugar levels back to normal. The most important of those counter-regulatory hormones are glucagon, which is produced in pancreatic alpha cells, and epinephrine, which is secreted from the adrenal glands. Other hormones involved in the counter-regulatory response include growth hormone, which is produced in the pituitary gland, and cortisol, which is produced in the adrenal glands. (For more information on those hormones and their functions, see the article by Hiller-Sturmhöfel and Bartke, pp. 153–164.) With increasing duration of diabetes, however, the counter-regulatory responses of first glucagon and then epinephrine wane, rendering the diabetic patient more vulnerable to severe hypoglycemia, whether it results from medications or other causes.

Two additional medications—metformin and troglitazone—are now being used to treat people with type 2 diabetes. These agents act to lower the patient’s blood sugar levels by decreasing insulin resistance rather than by increasing insulin secretion. Accordingly, these medications help control blood sugar levels without causing hypoglycemia.

## Alcohol’s Effects on Blood Sugar Levels of Diabetics

Numerous studies have investigated alcohol’s effects on the control of blood sugar levels in diabetics. Those effects differ substantially depending on whether alcohol consumption occurs when the person has just eaten and blood sugar levels are relatively high (i.e., in the fed state) or when the person has not eaten for several hours and blood sugar levels are relatively low (i.e., in the fasting state).

### Effects of Alcohol Consumption in the Fed State

In people with either type 1 or type 2 diabetes, single episodes of alcohol consumption (i.e., acute alcohol consumption) generally do not lead to clinically significant changes in blood sugar levels. In fact, some studies have indicated that isolated episodes of drinking with a meal may have a beneficial effect by slightly lowering blood sugar levels that tend to rise too high in diabetics (Swade and Emanuele 1997). This potentially beneficial effect was observed in both men and women, regardless of age. The alcohol amounts administered in those studies were usually between 0.5 g/kg (gram per kilogram body weight) and 1 g/kg, leading to blood alcohol levels (BALs) between approximately 0.03 and 0.1 percent^2^ (McDonald 1980). Those doses are equivalent to approximately 2.5 to 5 standard drinks.^3^ Interestingly, studies of acute alcohol exposure in nondiabetic people have yielded quite variable results, noting decreases, increases, or no changes in glucose levels.

Conversely, research has indicated that long-term (i.e., chronic) alcohol consumption in well-nourished diabetics results in increased blood sugar levels (i.e., hyperglycemia). For example, Ben and colleagues (1991) compared the blood sugar levels of 46 type 2 diabetics who were “habitual drinkers”; 35 nondrinking type 2 diabetics; and 40 nondiabetic, nondrinking control subjects. The habitual drinkers consumed an average of 45 grams of pure alcohol, corresponding to approximately 3 to 4 standard drinks, per day. For the study, the participants were hospitalized for 7 days and received a standard hospital diet. The researchers evaluated the following biochemical markers, which can serve as indicators of blood sugar control:

- Blood sugar levels in the fasting state
- Hemoglobin A1c (HbA1c), a blood component that reflects blood sugar control over the past 2 to 3 months (the higher the HbA1c levels, the higher were the average blood sugar levels)
- C-peptide, a molecule that is produced together with insulin (because C-peptide is more stable than insulin, it can be detected longer in the blood and is frequently measured as a reflection of the average insulin secretion).

Based on those biochemical markers, the researchers found the following results:

- On the day of admission to the hospital and on the following day, the fasting blood sugar levels of the drinking type 2 diabetics were significantly higher than those of the nondrinking type 2 diabetics. On later days, no significant differences in fasting blood sugar levels existed between the two groups of diabetics.
- HbA1c levels (and, by extension, average blood sugar levels) were significantly higher in drinking type 2 diabetics than in nondrinking type 2 diabetics who, in turn, had significantly higher HbA1c levels than did the nondiabetic control subjects. Whether the elevated blood sugar levels in diabetics were caused directly by alcohol’s impact on blood sugar regulation or by the patients’ alcohol-related failure to comply with their diabetes treatment is unknown.
- C-peptide levels, and thus insulin production, were significantly lower in both groups of diabetics than in non-diabetics. No difference in C-peptide levels existed, however, between drinking and nondrinking diabetics, indicating that chronic alcohol consumption did not alter the diabetics’ insulin production. Consequently, the elevated glucose levels observed in the drinking diabetics likely were not caused by alcohol’s effects on insulin levels, but may have resulted from an alcohol-induced increase in insulin resistance in those diabetics.

The mechanism(s) underlying the increasing hyperglycemia in chronically drinking diabetics are still unknown. Because the most common form of diabetes, type 2 diabetes, is associated with both insufficient insulin secretion and insulin resistance, it appears likely that the alcohol-induced increase in blood sugar levels results from adverse effects on one or both of those variables. To date, no studies have specifically evaluated the effect of chronic alcohol ingestion on insulin secretion or insulin resistance, although the study described previously by Ben and colleagues (1991) suggests that chronic alcohol exposure does not diminish insulin secretion. Consequently, studies of the effects of acute alcohol ingestion currently provide the best means of assessing alcohol’s impact on insulin secretion and insulin resistance. Such experiments, which have been conducted mainly on non-diabetics, have demonstrated that acute alcohol administration leads to increased insulin resistance (Avogaro et al. 1983; Yki-Jarvinen and Nikkila 1985; Shelmet et al. 1988; Boden et al. 1993). In diabetic people, elevated insulin secretion cannot compensate for increased insulin resistance. The same mechanism might occur in chronically drinking diabetics and thus account for the deterioration of blood sugar control observed in those patients. Alternatively, chronically drinking diabetics may show worse compliance with their dietary and pharmacological treatment regimens, which also may result in uncontrolled blood sugar levels. Clearly, more studies in this area are needed.

### Effects of Alcohol Consumption in the Fasting State

In contrast to chronic alcohol consumption in the fed state—which raises blood sugar levels, resulting in hyperglycemia—alcohol consumption in the fasting state can induce a profound reduction in blood glucose levels (i.e., hypoglycemia). That effect has been observed in both type 1 and type 2 diabetics as well as in nondiabetics (Arky and Freinkel 1964). Hypoglycemia can have serious, even life-threatening, consequences, because adequate blood sugar levels are needed to ensure brain functioning.

Alcohol-induced hypoglycemia typically occurs in people (both diabetics and nondiabetics) who, sometimes for days, have been drinking alcohol but not eating. In such a fasting state, the body has two major mechanisms for maintaining the blood sugar levels necessary to provide energy to the brain: (1) breakdown of glycogen, or glycogenolysis, and (2) production of glucose, or gluconeogenesis.

Glycogen is a large molecule that consists of numerous glucose molecules and serves as a storage form of glucose in the tissues, particularly the liver. In the fasting state, as a first line of defense against hypoglycemia, glycogen is broken down into its constituent glucose molecules, which are secreted by the liver into the blood to maintain normal or near-normal blood sugar levels. Generally, the glycogen supply is depleted after 1 or 2 days of fasting. Thus, a person who has been drinking alcohol and not eating for 1 or more days has exhausted his or her glycogen supply.

Gluconeogenesis, which also occurs primarily in the liver, involves the formation of new glucose molecules from alanine and glycerol. Alanine is generated during the breakdown of proteins in the muscles, whereas glycerol is formed during the metabolism of certain fat molecules (i.e., triglycerides). Alcohol metabolism in the liver, however, actually shuts down the process of gluconeogenesis and thus the second line of defense against hypoglycemia. Consequently, both of the body’s mechanisms to sustain blood sugar levels are inactivated in people who consume alcohol but do not eat, resulting in profound hypoglycemia.

Two additional factors may further compound the risk of alcohol-related hypoglycemia and its associated consequences in alcohol-drinking but otherwise fasting people, particularly diabetics. First, alcohol consumption can lead to a situation called hypoglycemic unawareness in both diabetics and nondiabetics (Kerr et al. 1990). A hypoglycemic person normally experiences several warning symptoms, such as sweating, weakness, shakiness, nervousness, and pounding or racing of the heart. Diabetics in particular learn to recognize those symptoms and prevent a further decline in blood sugar levels by eating some food. A person in a state of hypoglycemic unawareness, however, may not notice or recognize those warning signs and is therefore at increased risk of severe hypoglycemia. Alcohol-related hypoglycemic unawareness likely results from the cognitive impairment that occurs when BALs reach 0.08 to 0.1 percent.

Second, diabetics who have consumed alcohol, particularly those with type 1 diabetes, experience a delayed glucose recovery from hypoglycemia. This means that after an episode of hypoglycemia, glucose levels return to normal more slowly in drinking diabetics than in nondrinking diabetics, suggesting an alcohol-related impairment in the counter-regulatory response to hypoglycemia (Avogaro et al. 1993). Detailed analyses demonstrated that although the glucagon and epinephrine responses to hypoglycemia were unaffected, the growth hormone and cortisol responses were reduced after alcohol consumption.

The combination of alcohol-induced hypoglycemia, hypoglycemic unawareness, and delayed recovery from hypoglycemia can lead to deleterious health consequences. For example, Arky and colleagues (1968) studied five diabetics who experienced severe hypoglycemia after ingesting alcohol. In all five patients, the alcohol-induced hypoglycemia induced neurological changes, such as incontinence, inability to follow simple commands, perseveration,^4^ disorientation, and impairment of recent memory. In three patients, those changes did not reverse, even after months or years. The two other patients died as a result of complications indirectly related to their hypoglycemia-induced neurological changes. Therefore, to avoid alcohol-related hypoglycemia and its consequences, diabetics should consume alcohol only with or shortly after meals.

## Alcohol’s Effects on Complications of Diabetes

### Diabetic Ketoacidosis

Ketoacidosis, which occurs primarily in diabetics, is a condition characterized by excessive levels of certain acids called ketone bodies (e.g., acetone, acetoacetate, and β-hydroxybutyrate) in the blood. Elevated levels of those compounds can cause nausea, vomiting, impaired mental functioning, coma, and even death. Ketoacidosis is caused by complete or near-complete lack of insulin and by excessive glucagon levels. Among their many functions, insulin and glucagon regulate the conversion of fat molecules (i.e., fatty acids) into larger molecules (i.e., triglycerides), which are stored in the fat tissue. In the absence of insulin, the triglycerides are broken down into free fatty acids, which are secreted into the bloodstream and delivered to the liver. The liver normally re-incorporates free fatty acids into triglycerides, which are then packaged and secreted as part of a group of particles called very low-density lipoproteins (VLDL). In patients with ketoacidosis, however, the liver metabolizes the incoming free fatty acids in an additional, unusual way. Under the influence of excess glucagon, some of the free fatty acids are converted to ketone bodies and secreted into the blood, causing severe health consequences.

Ketoacidosis typically occurs in patients with type 1 diabetes who completely lack insulin. In rare cases, however, the condition also may affect people with type 2 diabetes. In a milder form, ketoacidosis may even occur in people who are fasting. In those people, insulin levels are diminished, because the fasting has considerably lowered their blood sugar levels, thereby depriving the pancreas of its stimulus to produce and secrete insulin.

Heavy alcohol consumption (i.e., 200 grams of pure alcohol, or approximately 16 standard drinks, per day) can cause ketoacidosis in both diabetics and nondiabetics (Wrenn et al. 1991). People who consume those high amounts of alcohol typically have been drinking and not eating for days and/or have vomited or developed other illnesses from drinking. As a result, those patients frequently have very low blood sugar levels (although some people with alcoholic ketoacidosis have very high blood sugar levels, because the lack of insulin prevents glucose uptake from the blood into the tissues).

The mechanisms underlying the development of alcoholic ketoacidosis are complex. However, some typical contributing factors result in insulin lack and excess glucagon levels, thereby promoting the development of ketoacidosis. As mentioned earlier in this article, poor food intake can lead to depleted glycogen levels. Furthermore, continued alcohol metabolism results in diminished gluconeogenesis. Both the depletion of glycogen and diminished gluconeogenesis lead to lower blood sugar levels. As blood sugar falls, insulin secretion is reduced as well. Because insulin restrains glucagon secretion, lower insulin secretion allows increased glucagon secretion, setting the stage for the development of ketoacidosis. This situation can be amplified if the drinker vomits repeatedly. Vomiting can lead to dehydration and a reduced blood volume, which, in turn, increases the levels of certain stress hormones in the blood called catecholamines. Catecholamines further decrease insulin production and increase glucagon production. Accordingly, physicians who treat diabetics known to consume large amounts of alcohol must be aware of the risk of alcoholic ketoacidosis in those patients.

### Alterations of Lipid Metabolism

Abnormalities in the levels and metabolism of lipids are extremely common in people with either type 1 or type 2 diabetes and may contribute to those patients’ risk of developing cardiovascular disease (Durrington 1995). Alcohol consumption can exacerbate the diabetes-related lipid abnormalities, because numerous studies have shown that heavy drinking can alter lipid levels even in nondiabetics. Alcohol can induce several types of lipid alterations, including elevated triglyceride levels in the blood (i.e., hypertriglyceridemia), reduced levels of low-density lipoprotein (LDL) cholesterol, and elevated levels of high-density lipoprotein (HDL) cholesterol.

#### Elevated Triglyceride Levels

Hypertriglyceridemia is an important risk factor for cardiovascular diseases. Moreover, elevated triglyceride levels can cause severe inflammation of the pancreas (i.e., pancreatitis). In addition to being highly painful and potentially fatal, this inflammation may interfere with the production of insulin, thereby potentially worsening control of blood sugar levels and making hypertriglyceridemia a particularly serious complication in diabetics. Heavy drinking (i.e., more than 140 grams of pure alcohol, or approximately 12 standard drinks, per day) can cause alcohol-induced hypertriglyceridemia in both diabetics and nondiabetics (Chait et al. 1972). In fact, from a practical standpoint, heavy drinking should be considered as a possible contributing factor in all patients with hypertriglyceridemia. Abstinence from alcohol generally leads to normalization of the triglyceride levels, unless the person has an underlying genetic predisposition for hypertriglyceridemia.

Several mechanisms may contribute to alcohol-induced increases in triglyceride levels. First, alcohol likely stimulates the generation of VLDL particles in the liver, which are rich in triglycerides. Second, alcohol may inhibit VLDL particle breakdown. Third, alcohol may enhance the increase in triglyceride levels in the blood that usually occurs after a meal.

#### Reduced LDL Cholesterol Levels

LDL cholesterol is strongly related to cardiovascular disease and stroke and has been called “bad” cholesterol. Reduction of LDL cholesterol decreases a person’s likelihood of suffering a heart attack or stroke. LDL cholesterol levels tend to be lower in alcoholics than in nondrinkers (Castelli et al. 1977), suggesting that chronic alcohol consumption may have a beneficial effect on cardiovascular risk. However, Lin and colleagues (1995) reported that the LDL cholesterol in alcoholics exhibits altered biological functions and may more readily cause cardiovascular disease. The researchers found that the levels of vitamin E, an agent that in part is bound to LDL cholesterol and which may decrease the risk of cardiovascular disease, also are lower in alcoholics than in nonalcoholics. Those observations suggest that the reduced levels of vitamin E in alcoholics actually may have harmful long-term effects.

#### Elevated HDL Cholesterol Levels

HDL cholesterol has a protective effect against cardiovascular disease and is called “good” cholesterol. This protective effect results at least partly from a process called reverse cholesterol transport, in which HDL particles carry cholesterol from blood vessel walls and other sites back to the liver, where it is broken down and subsequently eliminated from the body. Two subtypes of HDL—HDL_2_ and HDL_3_—are particularly effective in this reverse cholesterol transport. Studies in alcoholics found that the levels of HDL, and particularly of HDL_2_ and HDL_3_, were elevated after a period of chronic drinking and returned to normal levels after several days of abstinence (Taskinen et al. 1982). Moreover, epidemiological data have demonstrated that moderate alcohol consumption of up to three standard drinks per day is associated with a reduced risk of heart attacks and that this effect is partly mediated by alcohol-induced increases in HDL_2_ and HDL_3_ levels (Gaziano et al. 1993).

### Cardiovascular Disease

Cardiovascular disease continues to be one of the leading causes of death among all Americans and is the leading cause of death in people with type 2 diabetes (Bierman 1992). The relationship of alcohol consumption to cardiovascular disease in diabetic people has not been well evaluated. However, substantial information on the association of alcohol and cardiovascular disease exists from population studies that included an unknown percentage of diabetics. For example, in one 10-year study of 1,422 men, overall death rates (and, by extension, death rates from cardiovascular disease) were lowest among those who consumed 0.1 to 34 grams of alcohol per day (i.e., between 1 standard drink every few months and approximately 3 standard drinks per day) (Marmot et al. 1981). Both complete abstainers and people drinking more than 34 grams of alcohol per day had higher death rates, with abstainers exhibiting the highest death rates from cardiovascular disease and heavy drinkers exhibiting the highest death rates from noncardiovascular disease. Those findings suggest that alcohol consumption, particularly moderate consumption, may have a protective effect against cardiovascular disease.

Alcohol consumption also can influence blood pressure. In population studies, moderate alcohol consumption of less than two drinks per day was associated with slightly lower blood pressure than abstinence, particularly among women (Harburg et al. 1980; Klatsky et al. 1977). Consumption of more than three drinks, however, resulted in elevated blood pressure in both men and women compared with nondrinkers. Because high blood pressure is a risk factor for cardiovascular disease, those results also suggest that moderate alcohol consumption can have beneficial effects for cardiovascular disease risk. It is unknown, however, whether that effect applies equally to diabetics and nondiabetics, because those studies did not specify the proportion of diabetic participants. The mechanisms underlying alcohol’s impact on blood pressure have not been fully elucidated.

### Peripheral Neuropathy

Peripheral neuropathy is a condition in which nerves are damaged that extend from the spinal cord to control muscle function (i.e., motor nerves) or that transmit various sensations—such as touch, pain, temperature, and vibration—back to the spinal cord and brain (i.e., sensory nerves). Although many patients do not experience symptoms of peripheral neuropathy, other patients suffer from varied and troublesome symptoms, such as tingling, burning, pain, and numbness. Those symptoms occur most commonly in the legs and feet and are frequently worse at night. Numbness in particular can be a serious problem: Patients may not sense lesions, such as cuts or ulcers, which may then become seriously infected and result in amputation of the affected limb.

Diabetes and alcohol consumption are the two most common underlying causes of peripheral neuropathy. Among diabetics, the prevalence of neuropathy with obvious symptoms (i.e., symptomatic neuropathy) increases with increasing disease duration. Moreover, McCulloch and colleagues (1980) reported that for any given duration of diabetes, the prevalence of symptomatic peripheral neuropathy was greater in men who consumed at least three to four alcohol-containing beverages almost every night compared with men who drank less. That increase in prevalence was most apparent in patients with a disease duration of less than 4 years. Other researchers observed that the prevalence of neuropathy in type 1 diabetics increased in a linear fashion with the alcohol amount consumed (Mitchell and Vinik 1987). Those researchers also reported that diabetics who consumed more than eight standard drinks per week developed peripheral neuropathy faster than did diabetics who consumed eight or fewer drinks per week.

Alcohol use also is associated with an inability to sense vibrations from a tuning fork (i.e., absent vibratory perception), which serves as a convenient semiquantitative measure of peripheral neuropathy (Sosenko et al. 1986). All of these findings suggest that alcohol and diabetes can enhance each other’s effects in terms of causing nerve damage. Because neuropathy is a major clinical problem in diabetics, more analyses are needed of the precise quantitative relationship between alcohol intake and neuropathy in diabetics as well as on factors that may reverse or protect against the neuropathy.

#### Impotence

Neuropathy, in addition to other factors (e.g., vascular disease in the penis or altered hormone levels), also may contribute to impotence, which is a common and troublesome complication in diabetic men. The nerves that control erection are part of the autonomic nervous system, which controls numerous vital processes that occur without conscious efforts (e.g., breathing and the contractions of the gut necessary for proper digestion).

Despite the high prevalence of impotence in male diabetics and the fact that many of these men consume alcohol, few studies have evaluated the relationship between alcohol intake and impotence in diabetics. In one study of 275 originally potent diabetic men, heavy drinkers were significantly more likely to develop impotence during the 5-year study period than were moderate drinkers (McCulloch et al. 1984). Based on assumptions regarding the alcohol content of the beverages mentioned in the study, “heavy” drinkers were defined as those who ingested 29 grams of alcohol, or approximately two to three standard drinks, per day.

Many impotent diabetic men also have lower than normal levels of the sex hormone testosterone in their blood. Alcohol reduces blood levels of testosterone and may thereby further exacerbate the existing hormonal deficit. Clinical experience indicates, however, that a testosterone deficit rarely is the sole reason for impotence in diabetic men, because treatment with testosterone rarely restores potency in those men. Thus, both neuropathy and vascular disease likely play significant roles in impotence in diabetic men.

### Retinopathy

Diabetic eye disease (i.e., retinopathy) is another troublesome tissue complication of diabetes and one of the leading causes of blindness in the United States today. Good blood sugar and blood pressure control as well as regular eye examinations are essential for the prevention of retinopathy. Heavy alcohol consumption may increase a person’s risk for developing this disease. Thus, in one study of 296 male diabetics who had no eye disease at the beginning of the study, those men who drank the equivalent of more than 10 pints of beer per week were more likely to develop retinopathy over 5 years than were those men who drank less or were abstainers (Young et al. 1984). Interestingly, the risk of retinopathy was independent of the men’s ability to control their blood sugar, suggesting that alcohol may directly damage the eyes or related structures.

The much larger United Kingdom Prospective Diabetes Study confirmed that alcohol consumption in men (but not in women) was associated with more severe retinopathy (Kohner et al. 1998). The relationship between alcohol consumption and retinopathy in that study, however, was not independent (i.e., it may have resulted from other intermediary factors, such as greater alcohol-related blood sugar elevation in drinking diabetics than in nondrinking diabetics). Consequently, the information regarding the relationship between alcohol ingestion and diabetic eye disease remains inconsistent, underlining the need for further studies.

### Medication Interactions

In addition to exacerbating various medical complications of diabetes, alcohol consumption and its associated health consequences may interact with or alter the effects of several medications used to treat diabetes, including the following:

- Chlorpropamide is a medication used to treat type 2 diabetes by increasing pancreatic insulin secretion. Some people treated with chlorpropamide experience an unpleasant, disulfiram-like reaction^5^ after drinking alcohol.
- Metformin, a medication that decreases insulin resistance, can cause potentially lethal side effects in patients whose liver is not functioning properly. Accordingly, patients who abuse alcohol and are therefore at risk for liver damage must not take metformin.
- Troglitazone, another medication that decreases insulin resistance, also must not be used by patients with liver disease and therefore should not be used by alcohol abusers. Moreover, troglitazone itself may impair liver function, and alcohol might further exacerbate this harmful effect.

## Summary

Occasional episodes of alcohol consumption generally do not worsen blood sugar control in people with diabetes and may even have beneficial effects. Regular consumption of even moderate amounts of alcohol (i.e., two to four drinks per day), however, clearly interferes with diabetic blood sugar control and increases the risk of impotence; peripheral neuropathy; and, possibly, retinopathy. At the same time, similar levels of alcohol consumption are associated with a decreased risk of heart attacks and death from cardiovascular disease. The latter findings, however, were obtained with populations that included diabetics as well as nondiabetics, thereby limiting researchers’ ability to apply those findings to diabetics.

Accordingly, more studies are needed to determine whether the beneficial effects of daily moderate alcohol consumption outweigh the deleterious effects. Diabetics clearly should avoid heavy drinking (i.e., more than 10 to 12 drinks per day), because it can cause ketoacidosis and hypertriglyceridemia. Moreover, heavy drinking in a fasting state can cause hypoglycemia and ultimately increase diabetics’ risk of death from noncardiovascular causes.

## Footnotes

1 The prefix “hyper-” always indicates higher than normal levels of a substance, whereas the prefix “hypo-” indicates lower than normal levels. The suffix “-emia” refers to the levels of a substance in the blood. Thus, hyperinsulinemia refers to higher than normal insulin levels in the blood, whereas hypoglycemia refers to lower than normal glucose levels in the blood.

2 In most States, BALs of 0.08 to 0.1 percent are the legal limits for intoxication.

3 A standard drink contains 12 grams (approximately 0.5 ounce) of pure alcohol. This amount is equal to one 12-ounce bottle of beer or wine cooler, one 5-ounce glass of wine, or 1.5 ounces of distilled spirits.

4 Perseveration is the involuntary repetition of words or actions.

5 Disulfiram (Antabuse^®^) is a medication used to treat alcoholics. It acts by inducing an unpleasant physical response (e.g., nausea and vomiting) after alcohol consumption.
